# Reply to “Genome of *Rhodovulum iodosum*, a marine photoferrotroph”

**DOI:** 10.1128/mra.01088-24

**Published:** 2025-07-29

**Authors:** Arpita Bose, Dinesh Gupta, Casey Bryce, Andreas Kappler

**Affiliations:** 1 Department of Biology, Washington University in St. Louis, St. Louis, Missouri, USA; 2 Department of Molecular and Cellular Biology, University of California at Berkeley, Berkeley, California, USA; 3 School of Earth Sciences, University of Bristol, Bristol, United Kingdom; 4 Department of Geosciences, University of Tubingen, Tubingen, Germany; University of Maryland School of Medicine, Baltimore, Maryland, USA

## REPLY

This is a reply to "Genome of *Rhodovulum iodosum*, a marine photoferrotroph" (1). We (Arpita Bose and Dinesh Gupta) acquired *Rhodovulum robiginosum* DSM 12329^T^ from the Leibniz-Institut DSMZ GmbH in 2018 for genome sequencing. We subsequently genome sequenced the strain that we acquired and published this as “Draft Genome Sequence of a Marine Photoferrotrophic Bacterium, *Rhodovulum robiginosum* DSM 12329^T^” in *Microbiology Resource Announcements* in February 2019 (2). New analysis now shows that our genome for *Rhodovulum robiginosum* DSM 12329^T^ does not share the same 16S rRNA gene as the original *Rhodovulum robiginosum* published by Straub et al. (3). Instead, it is identical to the *Rhodovulum iodosum* isolated in the same study on both the 16S rRNA gene and whole genome level. Based on this assessment, we now believe that *R. robiginosum* DSM 12329^T^ has likely been swapped with *R. iodosum* DSM 12328^T^ by the Leibniz-Institut DSMZ GmbH and that our genome is most likely that of *R. iodosum* DSM 12328^T^.

Since the time of our analyses, the Leibniz-Institut DSMZ GmbH has added notices to both *R. robiginosum* DSM 12329^T^ and *R. iodosum* DSM 12328^T^, indicating discrepancies in the 16S rRNA gene sequence, which indicate that the 16S rRNA gene sequence for each of the strains is, in fact, identical to the other strain. In short, they are aware of this swap. We hope that highlighting these issues here will prevent others from pursuing studies with the wrong microorganism.

Because the Leibniz-Institut DSMZ GmbH, which distributes both *R. iodosum* DSM 12328^T^ and *Rhodovulum robiginosum* DSM 12329^T^ strains, has not corrected the likely microbial strain swap, we are writing this reply in coordination with the authors of the Microbial Resource Announcements “Genome of *Rhodovulum iodosum*, a marine photoferrotroph” in particular Casey Bryce and Andreas Kappler to ensure that (i) we are all aware of the strain swap that likely occurred at Leibniz-Institut DSMZ GmbH, and (ii) we can ensure that others who acquire these strains from the Leibniz-Institut DSMZ GmbH vs. from the Kappler laboratory are aware of this issue. Finally, we hope that this clarification encourages the community that studies these microorganisms and accesses their genomic information from various databases to use caution, and independently validate the identity of these strains using approaches such as 16S rRNA gene sequence comparison.
